# Supplementary figures and images for: A replicon RNA vaccine can induce durable protective immunity from SARS-CoV-2 in nonhuman primates after neutralizing antibodies have waned
Source: PLoS Pathog. 2023 Apr 19;19(4):e1011298. doi: 10.1371/journal.ppat.1011298 (PMC10150980; doi:10.1371/journal.ppat.1011298)

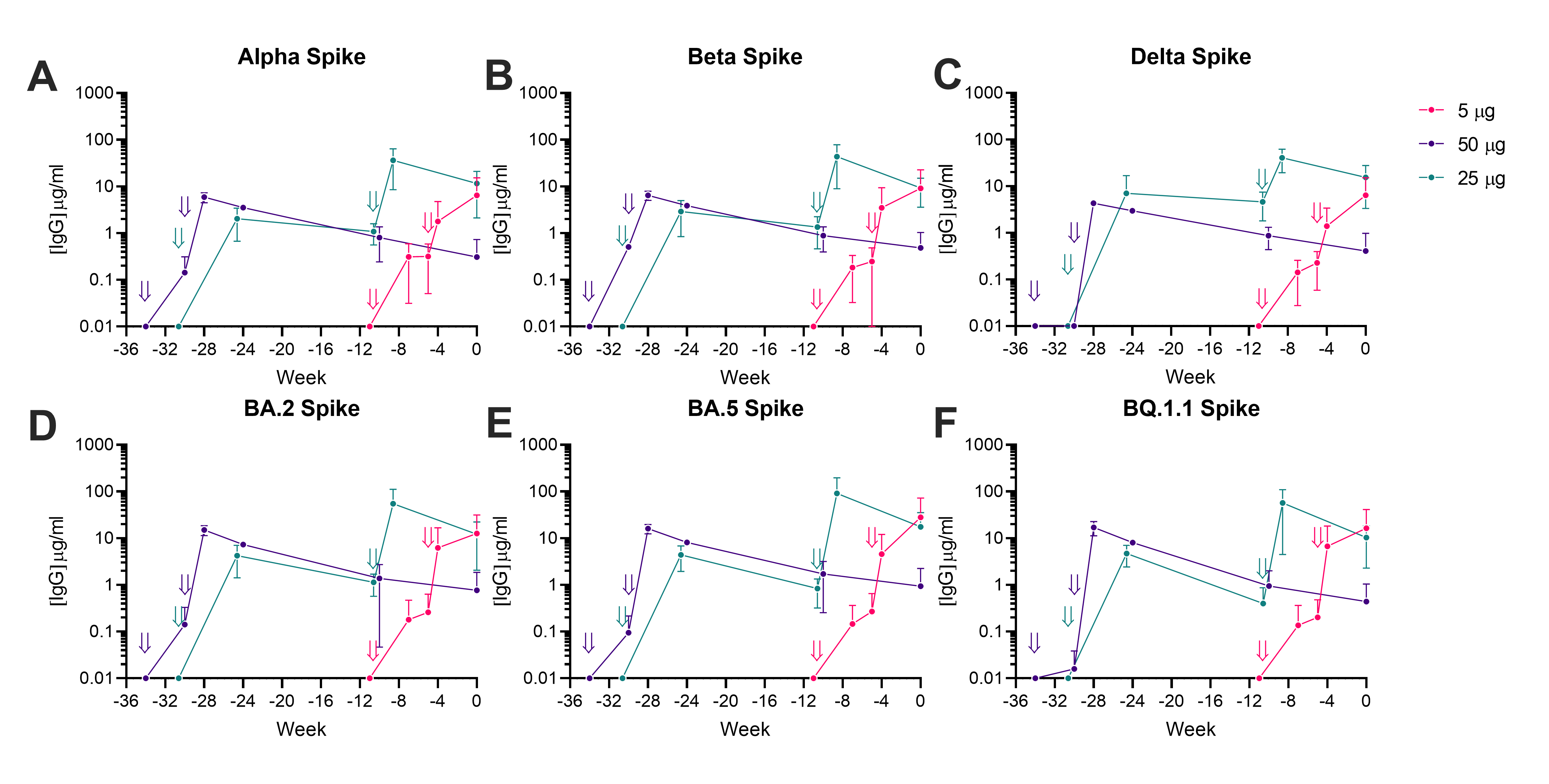

Supplement: S1 Fig — Serum anti-S (A) Alpha, (B) Beta, (C) Delta, (D) BA.2, (E) BA.5, and (F) BQ.1.1 titers as determined by ELISA. (A-F) Means and standard deviations are shown. (TIF) [file ppat.1011298.s001.tif]

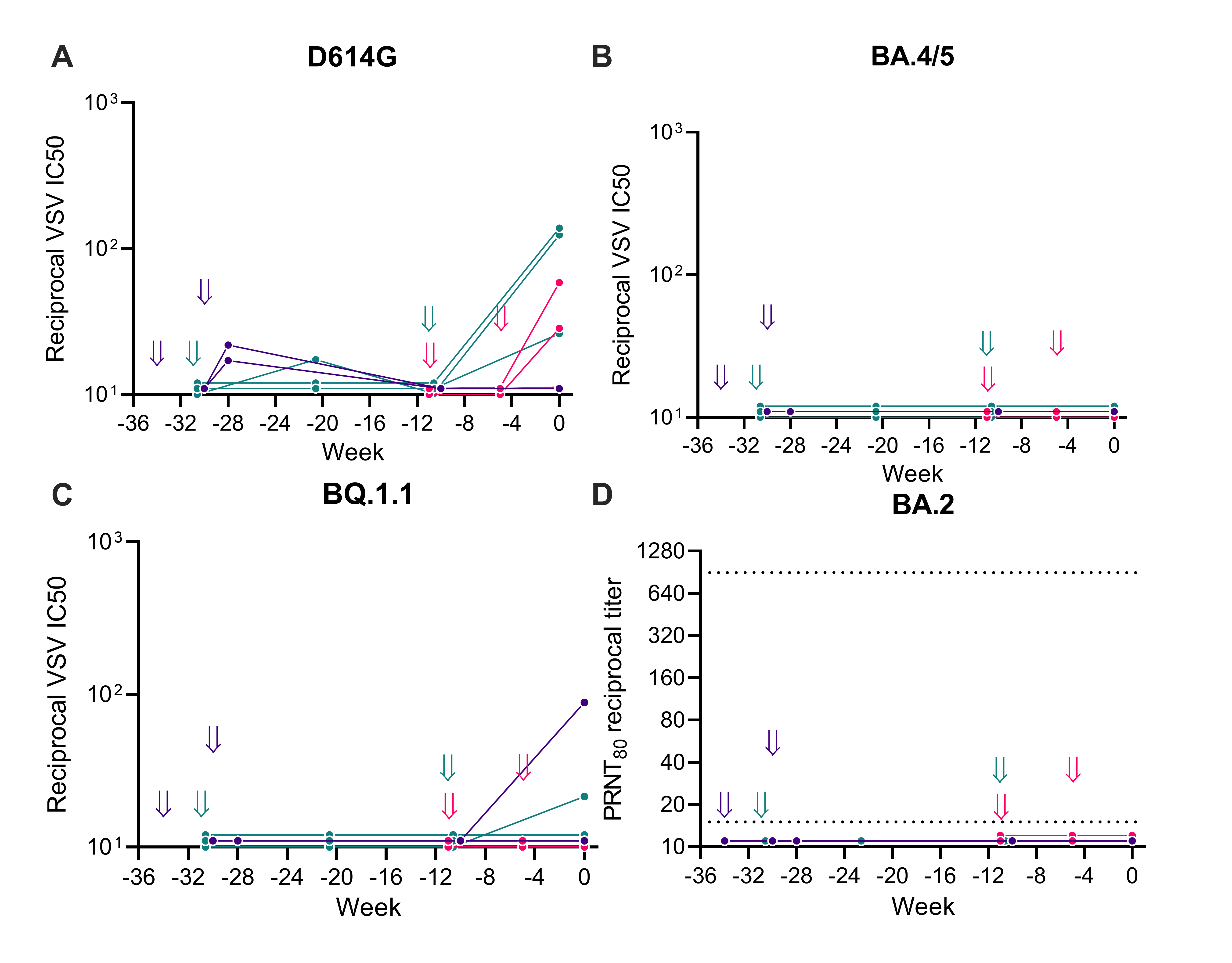

Supplement: S2 Fig — Neutralizing antibody titers against VSV pseudotyped viruses harboring (A) D614G, (B) BA.4/5, or (C) BQ.1.1 SARS-CoV-2 Spike. (D) Neutralizing antibody titers measured by PRNT80 against Omicron BA.2 SARS-CoV2 isolate. (TIF) [file ppat.1011298.s002.tif]

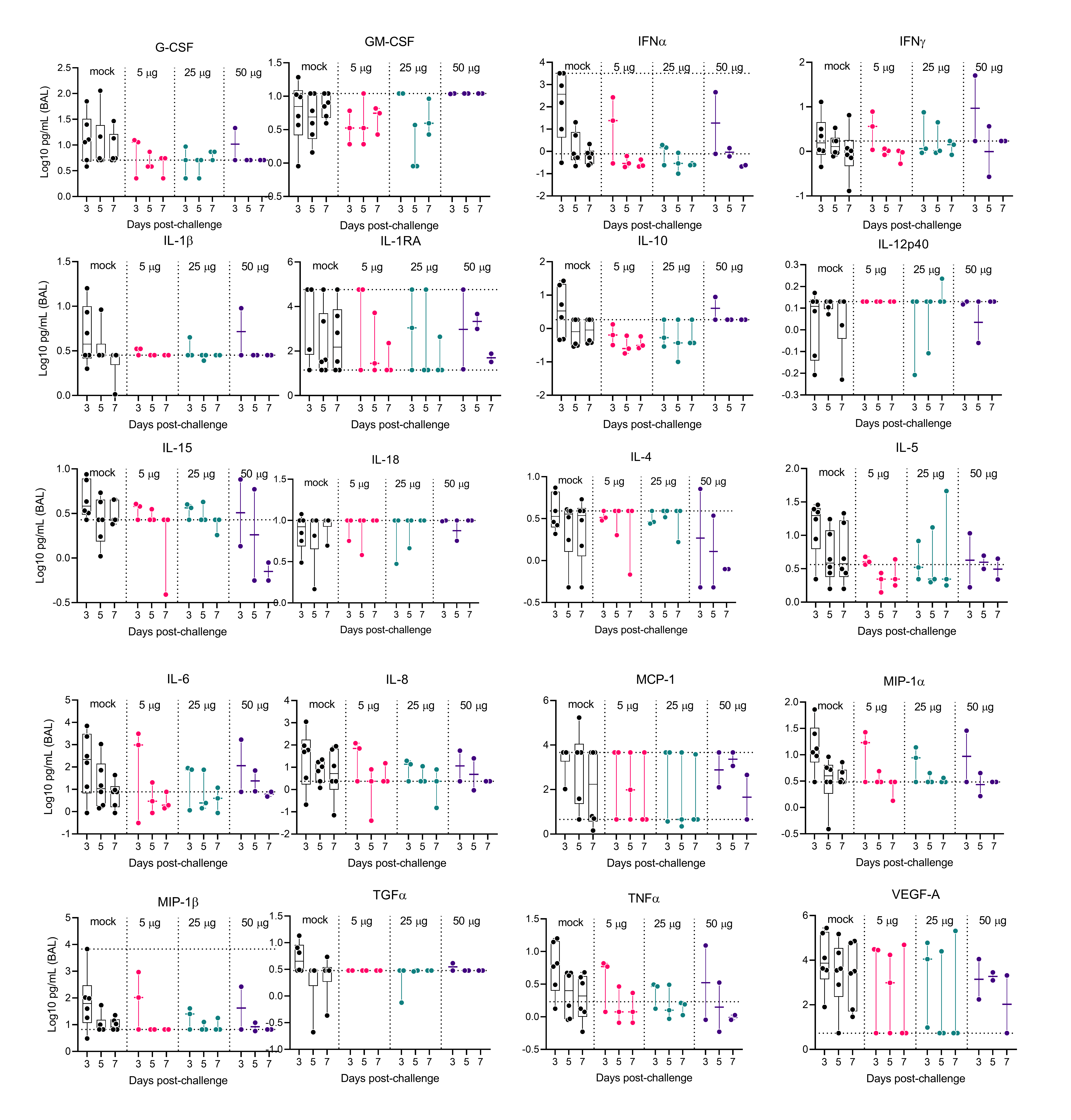

Supplement: S3 Fig — Shown are log transformed concentrations of cytokines and chemokines measured in BAL by multiplex immunoassay. Horizontal dotted lines represent the lower (LLOQ) and upper (ULOQ) limits of quantification for the assay. (TIF) [file ppat.1011298.s003.tif]

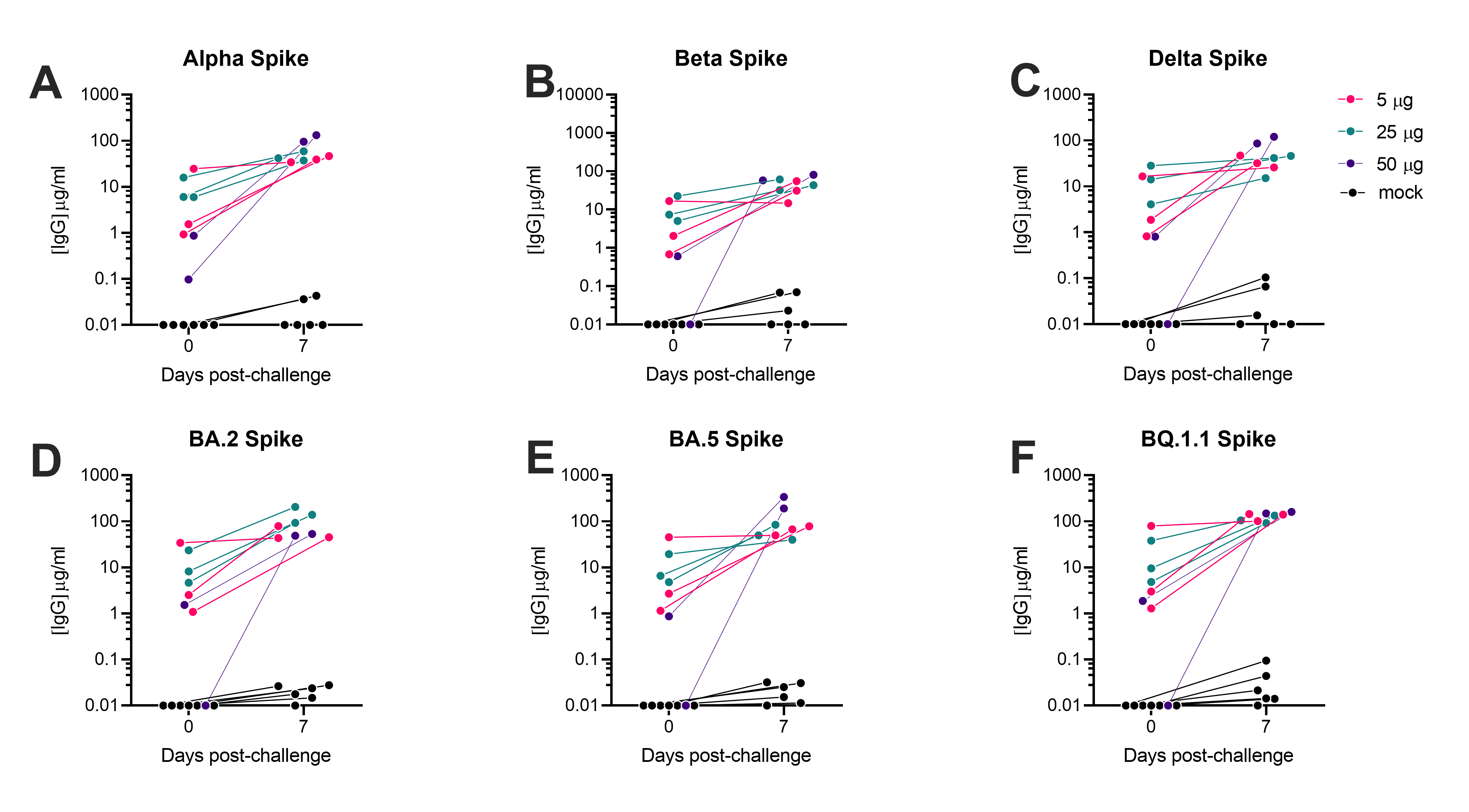

Supplement: S4 Fig — Serum anti-S (A) Alpha, (B) Beta, (C) Delta, (D) BA.2, (E) BA.5, and (F) BQ.1.1 titers as determined by ELISA. (A-F) Means and standard deviations are shown. (TIF) [file ppat.1011298.s004.tif]

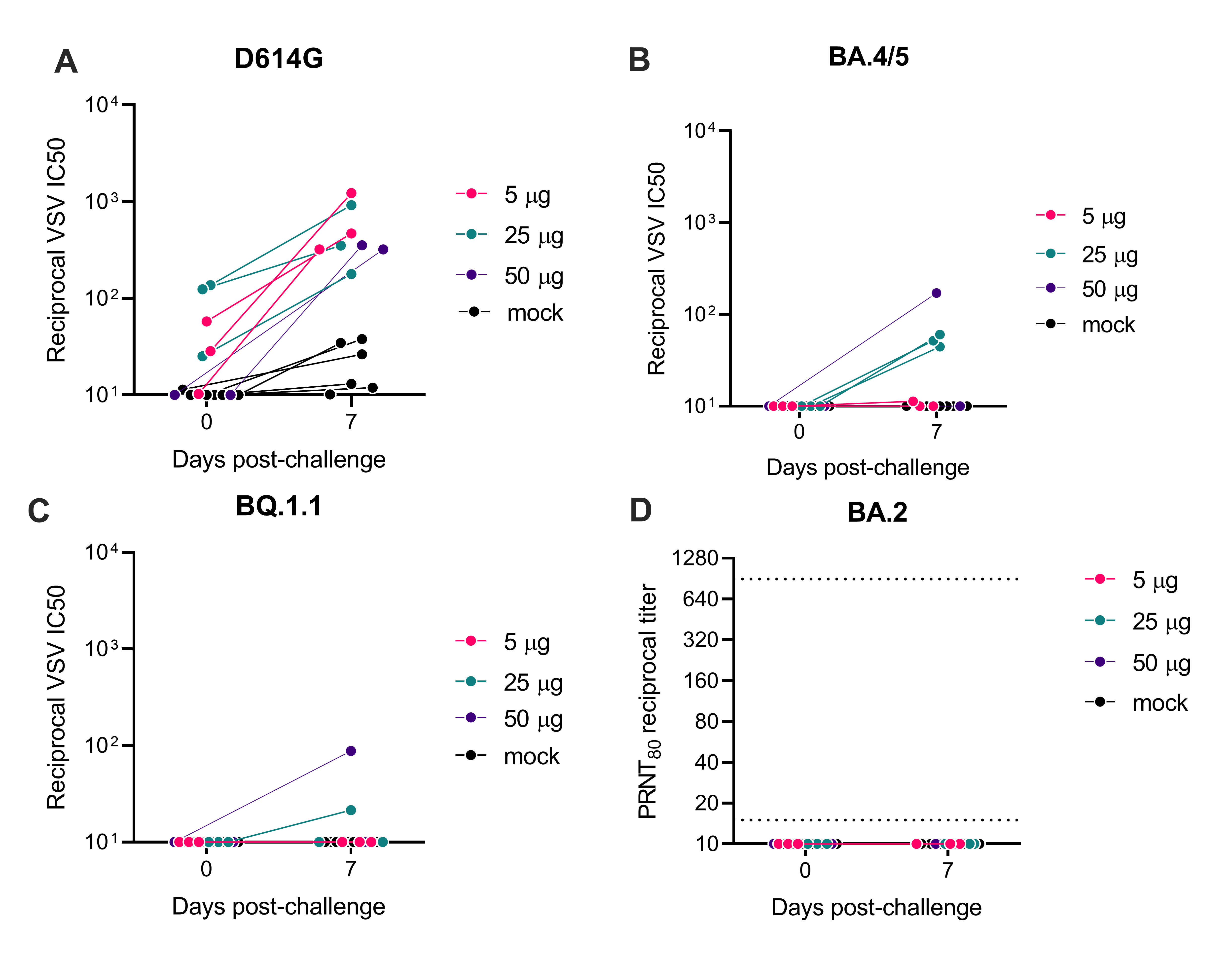

Supplement: S5 Fig — Neutralizing antibody titers against VSV pseudotyped viruses harboring (A) D614G, (B) BA.4/5, or (C) BQ.1.1 SARS-CoV-2 Spike. (D) Neutralizing antibody titers measured by PRNT80 against Omicron BA.2 SARS-CoV2 isolate. (TIF) [file ppat.1011298.s005.tif]
